# Introduction of a CMR-conditional cardiac phantom simulating cardiac anatomy and function and enabling training of interventional CMR procedures

**DOI:** 10.1038/s41598-019-56506-8

**Published:** 2019-12-27

**Authors:** Michael Bietenbeck, Anca Florian, Grigorios Chatzantonis, Claudia Meier, Dennis Korthals, Sven Martens, Ali Yilmaz

**Affiliations:** 1 Department of Cardiology, Division of Cardiovascular Imaging, University Hospital Münster, Münster, Germany; 2 Department of Cardiac Surgery, University Hospital Münster, Münster, Germany

**Keywords:** Interventional cardiology, Preclinical research

## Abstract

Interventional magnetic resonance imaging (MRI) procedures promise to open-up new vistas regarding clinically relevant diagnostic and/or therapeutic procedures in the field of cardiology. However, a number of major limitations and challenges regarding interventional cardiovascular magnetic resonance (CMR) procedures still delay their translation from pre-clinical studies to human application. A CMR-conditional cardiac phantom was constructed using MR-safe or -conditional materials only that is based on a unique modular composition allowing quick replacement of individual components. A maximal flow of 76 ml/sec in the aorta and 111 ml/sec in the pulmonary artery were measured, whereas the maximal flow velocity was 56 cm/sec and 89 cm/sec, respectively. A conventional wedge-pressure catheter was advanced over a MRI-conditional guidewire into the right ventricle and thereafter positioned in the pulmonary artery. Pulmonary artery pressure was measured, obtaining the following values for our cardiac phantom: max/min/mean = 16/10/12 mmHg. The presented CMR-conditional cardiac phantom is the first of its kind that does not only mimic cardiac mechanics with adjustable fluid pressure in a four chamber setup that is closely adapted to that of the human heart, but also enables introduction and testing of interventional tools such as guidewires and catheters.

## Introduction

Interventional magnetic resonance imaging (MRI) procedures promise to open-up new vistas regarding clinically relevant diagnostic and/or therapeutic procedures in the field of cardiology. On the one hand, cardiovascular magnetic resonance (CMR) enables a detailed visualization of cardiac anatomy and pathology without any radiation burden, but it also enables the detection and monitoring of post-procedural changes. This allows almost real-time (RT) assessment of the procedures’ success. In view of these advantages (pre-)clinical research is increasing. A number of interventional CMR procedures have already been successfully performed in smaller human studies such as right heart catheterization^1–3^ or electrophysiological CMR-guided radiofrequency (RF) ablation of atrial flutter^4–6^. In addition, RT-CMR-guided endomyocardial biopsy has only been tested *ex vivo* or in animal models so far^7–10^.

Traditionally, pre-clinical studies using large animals such as swine are a good approach to model the human cardiovascular system – and allow testing of interventional CMR procedures as well as biocompability of tools. However, increasing costs, legal regulations and ethical implications limit large animal studies and thereby may delay the introduction of interventional CMR to the clinic. As a consequence, these challenges result in (a) limited CMR-conditional materials and devices (such as guidewires, guiding sheaths, bioptomes, ablation catheters, etc.), (b) limitations in appropriate RT-CMR sequences regarding the needed temporal and spatial resolution and (c) lack of experience due to missing of opportunities for training researchers and clinicians interested in interventional CMR.

Hence, the aim of the present study was to develop a CMR-conditional cardiac phantom that simulates both, cardiac anatomy and function, and to demonstrate the phantom’s capabilities in training interventionalists in procedures such as CMR-guided right heart catheterization.

## Methods

### CMR-conditional cardiac phantom

The presented prototype of a CMR-conditional cardiac phantom was developed in collaboration with Philipp Daum (Ing. Ph. J. Daum GmbH & Co. KG). It is built of MR-safe or -conditional materials in a unique modular composition that allows quick replacement of individual components in case of need (e.g. after destruction during a training procedure). Similar to a human heart, this phantom comprises two atrial and ventricular chambers that are linked by four cardiac valves (bioprostheses). The elastic ventricular wall consists of a mixture of polyurethane. Systolic contraction of the symmetrically designed left and right ventricles is realized by external air pressure causing movement of an elastic membrane that in turn compresses fluid in the chamber adjacent to the ventricles. Consequently, this external pressure is transmitted to the ventricular walls causing systolic contraction. Diastolic relaxation is realized by interrupting the air pressure inflow (by use of a remote control unit) and elastic rebounce of the polyurethane walls. Elastic plastic tubes were used to simulate the vena cava inferior, the pulmonary artery, the (single) pulmonary vein (that was directly connected to the pulmonary artery tube) and the aorta.

### Technical aspects of CMR-guided right heart catheterization

A 5 F Arrow® wedge-pressure catheter from Teleflex (Ballon Wedge-Pressure Catheter, Teleflex, Pennsylvania, USA) was used for right heart catheterization. It was either filled with a gadolinium-based compound or air, and was advanced over an MRI-conditional guidewire from EPflex (MRLine, 0.035″, EPflex, Dettingen an der Erms, Germany). This guidewire is characterized by a good steerability and four metal markers at the distal end that enable visualization and targeted navigation.

### CMR protocol

CMR studies were performed on a 1.5-T Philips scanner with cardiac-dedicated surface coils (Ingenia, Best, The Netherlands). As outlined elsewhere^7^, the MR scanner was equipped with a dedicated intervention/navigation software platform called iSuite from Philips Research (Hamburg, Germany) enabling real-time imaging (RTI). The interventional operator is located in the scanner room, while the CMR operator is located outside in the control room. Communication between both operators and the conductance of the procedure itself is assured by interventional in-room monitors, speakers and microphones. A MR-conditional foot pedal can be used by the interventionalist to activate RTI-sequences. Re-adjustment as well as selection of modified slices is handled by the CMR-operator via the iSuite platform. During the entire MR examination, a fixed heart rate of 60 bpm was set/simulated on the scanner side. The external pump that drives the fluid air/fluid pressure for the systolic contraction allows to freely set the respective systolic and diastolic cyle length via a remote control unit and was set to a total cycle length approximately matching the simulated heart rate.

First, roadmap images were obtained using a 3D-balanced-SSFP sequence (TR = 1.94 ms, TE = 1.0 ms, FA = 45°, voxel size: 0.95 × 0.95 × 2 mm^3^). These roadmap images were transferred to the iSuite DICOM network node. Thereafter, iSuite was used to pre-define the required 2D imaging planes in order to perform a right heart catheterization. RTI was performed using those pre-planned imaging planes. The subsequent interventional procedure was done under RTI using a single-shot-balanced-SSFP-sequence (reconstructed voxel size: 1.25 × 1.25 × 8 mm³; temporal resolution: up to 0.2 s; TFE dur. shot/acq (ms): 316/253). Flow measurements were performed at the proximal level of the aorta and pulmonary artery by velocity-encoded (VENC) phase-contrast imaging (max. VENC: 150 cm/s, retrospective ECG‐triggering, phases per cardiac cycle: 30, repetition time: 3.9 ms, echo time: 2.4 ms, flip angle: 15°, field of view: 250 × 250 mm², acquisition matrix: 116 × 109, reconstructed voxel size: 1.3 × 1.3 × 10 mm³).

## Results

### Characteristics of the CMR-conditional cardiac phantom

**Figure 1:**
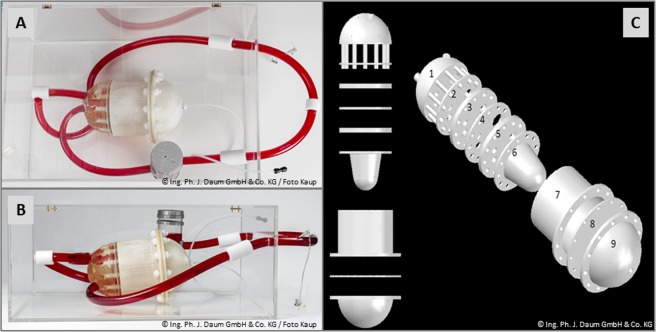
Pictures of our cardiovascular magnetic resonance (CMR)-conditional cardiac phantom, top view (**A**) and side view (**B**). Additional illustration of the unique modular composition that allows quick replacement of individual components in case of need (**C**).

The design and composition of our CMR-conditional cardiac phantom are illustrated in Fig. 1. The intracardiac and -vascular compartment (corresponding to the human intravasal compartment) were filled with a saline solution. The outer compartment (adjacent to the ventricular walls) that transmitted the external air pressure onto the ventricular walls was filled with a more dense solution (a mixture of agar, polyacrylamide and saline).

**Figure 2:**
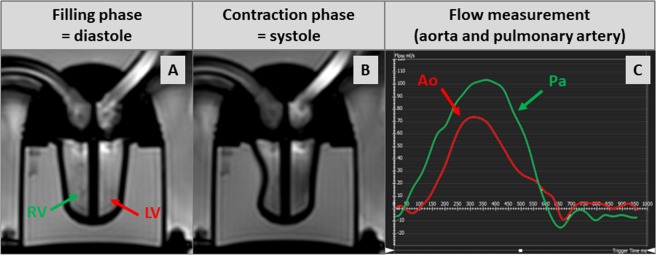
Cine-images of our cardiovascular magnetic resonance (CMR)-conditional cardiac phantom in a four-chamber view in diastole (**A**) and systole (**B**); respective flow measurement curves at the level of the plastic tubes simulating aorta and pulmonary artery (**C**).

A typical example of systolic contraction and diastolic relaxation of this phantom can be seen in Fig. 2A,B and Movie A1. According to our symmetrical design, left ventricular end-diastolic volume (LV-EDV) and right ventricular (RV)-EDV were similar: 42 ml. Ejection fraction (EF) depends on the chosen time interval for systole and diastole, respectively, and both can be freely chosen (within in a certain range) by use of the remote control unit. In the example presented here **(**Movie A1**)**, LV-EF was 20% and RV-EF was 40%. Minor differences in stroke volume between the left and right ventricle were due to minor differences in ventricular wall composition, but did not affect or limit the basic functioning of this phantom since an additional equalization chamber was integrated into the circulation.

Flow measurements using VENC-based phase-contrast imaging were performed at the level of the plastic tubes simulating aorta and pulmonary artery, respectively, and resulted in a maximal flow of 76 ml/sec in the aorta and 111 ml/sec in the pulmonary artery whereas the maximal flow velocity was 56 cm/sec in the aorta and 89 cm/sec in the pulmonary artery **(**Fig. 2C and Movie A2**)**.

The phantom was used several times (with more than two hours of pumping during each testing session and a total pumping time of more than 20 hours so far) and proved to be (a) MRI conditional at 1.5-T without any safety issues, (b) highly stable and robust without any destruction of individual components including the elastic ventricular walls consisting of polyurethane and (c) well suited for training in the use of interventional navigation platforms but also for initial tests of interventional procedures without any time constraints.

### CMR-guided right heart catheterization

**Figure 3:**
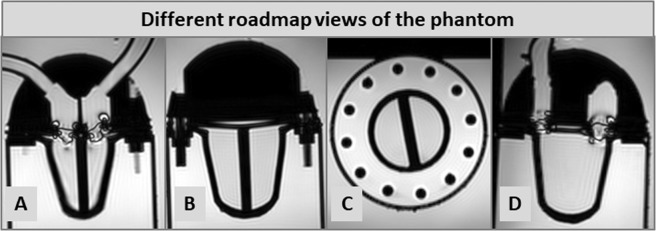
Different roadmap views that were used for subsequent real-time imaging (RTI)-guided right heart catheterization: (**A**) long-axis four-chamber view, (**B**) long-axis view of the ventricles at their maximal width, (**C**) mid-ventricular short-axis view and (**D**) long-axis right ventricular out-flow tract view.

Prior to the interventional procedure, roadmap images were obtained in different cardiac views. These were used for planning but also for orientation aid during right heart catheterization using a 3D-balanced-SSFP sequence **(**Fig. 3**)**. By the help of the previously defined views, the introduction of the guidewire and the wedge-pressure catheter into the right atrium, the right ventricle and the pulmonary artery can be tracked passively. A (artificial) femoral access site was chosen by the interventional operator in front of the MR scanner (on the right side of the phantom). Considering MR-imaging, the temporal resolution of RTI was up to 0.2 sec, equivalent with a frame rate of approx. 4–5 frames/sec for one slice during the automatic, continuous imaging mode. This rather low frame rate forced the interventionalist to advance the guidewire and wedge-pressure catheter rather slowly and gently.

**Figure 4:**
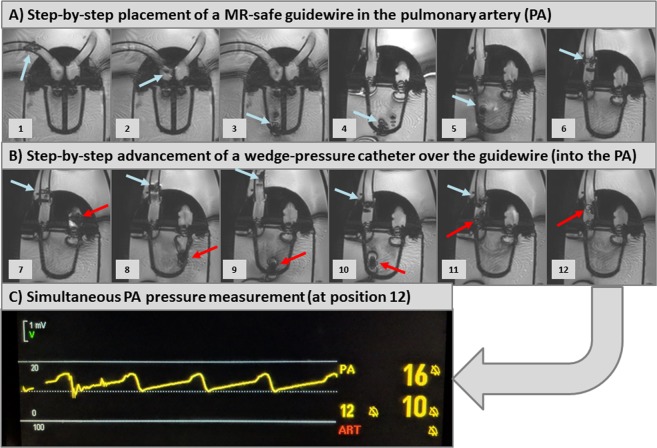
Step-by-step illustration of simulated right heart catheterization with our cardiovascular magnetic resonance (CMR)-conditional cardiac phantom: Panel A illustrates advancement of the MRI-conditional guidewire (blue arrow) through the right atrium, right ventricle into the pulmonary artery (PA). Panel B illustrates the advancement of the wedge-pressure catheter (red arrow) over the guidewire into the PA. Panel C shows the respective pressure curve that was documented after the wedge-pressure catheter entered the PA.

First, the MRI-conditional guidewire was advanced into the right ventricle **(**Fig. 4A and Movie B1–2**)** and was steered into the pulmonary artery **(**Fig. 4A and Movie C1–2). A conventional wedge-pressure catheter was then advanced over the guidewire into the right ventricle **(**Fig. 4B and Movie D) and thereafter positioned in the pulmonary artery **(**Fig. 4B and Movie E1–2**)**. Thereafter, pressure-measurements were performed at the level of the pulmonary artery **(**Fig. 4C**)** using the wedge-pressure catheter connected to an external amplifier (located in the control room): max/min/mean = 16/10/12 mmHg.

The entire RV catheterization took approx. 15 min including several re-runs and re-adjustments for the training of the CMR technologists and the interventionalist. In doing so, a whole body SAR of 0.78 W/kg was calculated (the upper limit of the whole body SAR during a standard MRI examination in normal operation mode is <2 W/kg).

## Discussion

In the past, different groups and/or vendors developed and offered so-called MRI-conditional cardiovascular phantoms for MRI. However, none of those phantoms available so far do allow to perform/test interventional procedures in an approximately realistic pumping system reflecting the beating human heart and its challenging contractile physiology. The available cardiovascular phantoms comprise either silicone-based, rigid, non-pumping 3D-constructions^11^ or a simple pumping model that does not allow to introduce any guidewires, catheters, etc^12,13^. To the best of our knowledge, the presented CMR-conditional cardiac phantom is the first that does not only mimic cardiac mechanics with adjustable fluid pressure in a four chamber setup that is closely adapted to that of the human heart, but also enables the introduction and testing of interventional tools such as guidewires and catheters. Hence, this novel phantom is a welcome tool for training purposes regarding interventional CMR procedures such as CMR-guided right heart catheterization, endomyocardial biopsy and others.

The measured volumetric values of the presented CMR-conditional cardiac phantom (EDV = 42 ml, EF = 20–40%, stroke volume = 18 ml) are similar to those of a child’s heart. In principle, a bigger phantom with higher ventricular volumes can be easily realized based on our phantom design; however, the current size is sufficient for the training of procedures such as right heart catheterization, endomyocardial biopsy, transseptal puncture, closure of an atrial septal defect (ASD), atrial flutter ablation, etc. Our approach simulating a beating heart is based on external air pressure causing movement of an elastic membrane that in turn compresses fluid in the chamber adjacent to the ventricles. This additional pressure chamber enlarges both the longitudinal and radial diameter of our phantom and thereby increases the size of the field-of-view (FOV) required for RTI-CMR. Since a larger FOV is associated with a larger imaging matrix (if spatial resolution is/should be unchanged), temporal resolution decreases and RTI becomes rather challenging. To increase the temporal resolution by means of a smaller, required FOV, we intend to simplify the vascular compartment (corresponding to the human intravasal compartment) in the next generation of this phantom by decreasing the total width of our phantom. Obviously, interventional training procedures may and will lead to signs of wear and even destruction of some components. Considering relatively high costs of even simple silicone-based, rigid, non-pumping 3D-phantoms, no user can afford to damage or to replace such a phantom for training purposes frequently. The phantom presented here is characterized by a unique modular composition that allows for the easy replacement of individual (rather cheap) components by the user if there is any (un-)intended damage during training.

Right heart catheterization was chosen first by most groups that started performing interventional MRI since it is a simple procedure without a relevant risk of complication^1,2,14^. Noteworthy, MRI-guided right heart catheterization is already the standard procedure in some centers^2^. In the present study, successful right heart catheterization was performed using a MRI-conditional guidewire and a conventional wedge-pressure catheter. Our CMR-conditional cardiac phantom saved us from time-consuming and exhaustive applications for pre-clinical studies in animal models (that need to be approved by institutional animal care authorities) and allowed us (a) to gain sufficient experience in the use of the iSuite platform and (b) to test and modify our CMR sequences without any time constraints. Since other groups that are already performing MRI-guided right heart catheterization have documented a learning curve in procedure time^14^, our testing phantom may help to achieve both, to shorten/avoid complex animal studies and to accelerate the respective learning curves for a successful application in humans.

Noteworthy, pulmonary artery pressure measurements in our phantom resulted in pressure waveforms and values that are quite similar to those in humans. Future studies will allow us to simultaneously assess catheter-based pressure and MRI-based flow measurements at different disease-like settings in order to further optimize our MRI protocols for different constellations. Moreover, additional interventional procedures such as MRI-guided endomyocardial biopsy, transseptal puncture and/or ASD closure can already be tested with the present generation of our phantom.

### Limitations

Both atrial and ventricular chambers were designed symmetrically and therefore do not allow to address physiological/hemodynamic differences of systemic and pulmonary circulation. Moreover, our approach in simulating the beating heart (external air pressure causing movement of an elastic membrane that in turn compresses fluid in the chamber adjacent to the ventricles) does not allow to provoke essentially different hemodynamic conditions regarding the left and right ventricle. Furthermore, some artefacts were observed at the level of the cardiac valves (bioprostheses) that possibly originate from captured air in the mounting/surrounding of the valves. These artefacts prevented visualization of the opening as well as closure of the respective valves, but did not substantially influence the assessment of the ventricles.

## Conclusion

The presented CMR-conditional cardiac phantom is the first of its kind that does not only mimic cardiac mechanics with adjustable fluid pressure in a four chamber setup that is closely adapted to that of the human heart, but also enables introduction and testing of interventional tools such as guidewires and catheters. Hence, this novel phantom is a welcome tool for training purposes regarding interventional CMR procedures such as CMR-guided right heart catheterization, endomyocardial biopsy and others.

## Supplementary information

Movie-A1

Movie-A2

Movie-B1

Movie-B2

Movie-C1

Movie-C2

Movie-D

Movie-E1

Movie-E2

## Data Availability

The datasets used and/or analysed during the current study are available from the corresponding author on reasonable request.
