# Supplementary figures and images for: Introduction of a CMR-conditional cardiac phantom simulating cardiac anatomy and function and enabling training of interventional CMR procedures
Source: Sci Rep. 2019 Dec 27;9:19852. doi: 10.1038/s41598-019-56506-8 (PMC6934499; doi:10.1038/s41598-019-56506-8)

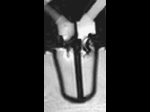

Supplement: Supplementary file 1 — Movie-A1 [file 41598_2019_56506_MOESM1_ESM.gif]

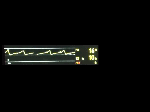

Supplement: Supplementary file 2 — Movie-A2 [file 41598_2019_56506_MOESM2_ESM.gif]

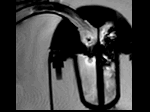

Supplement: Supplementary file 3 — Movie-B1 [file 41598_2019_56506_MOESM3_ESM.gif]

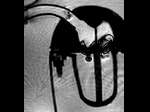

Supplement: Supplementary file 4 — Movie-B2 [file 41598_2019_56506_MOESM4_ESM.gif]

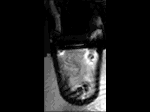

Supplement: Supplementary file 5 — Movie-C1 [file 41598_2019_56506_MOESM5_ESM.gif]

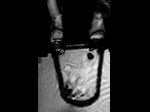

Supplement: Supplementary file 6 — Movie-C2 [file 41598_2019_56506_MOESM6_ESM.gif]

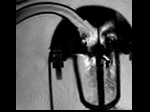

Supplement: Supplementary file 7 — Movie-D [file 41598_2019_56506_MOESM7_ESM.gif]

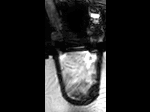

Supplement: Supplementary file 8 — Movie-E1 [file 41598_2019_56506_MOESM8_ESM.gif]

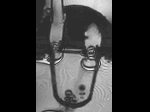

Supplement: Supplementary file 9 — Movie-E2 [file 41598_2019_56506_MOESM9_ESM.gif]
